# Distractor Dwelling, Skipping, and Revisiting Determine Target Absent Performance in Difficult Visual Search

**DOI:** 10.3389/fpsyg.2016.01152

**Published:** 2016-08-15

**Authors:** Gernot Horstmann, Arvid Herwig, Stefanie I. Becker

**Affiliations:** ^1^Department of Psychology, Bielefeld UniversityBielefeld, Germany; ^2^Cognitive Interaction Technology – Excellence Center, Bielefeld UniversityBielefeld, Germany; ^3^Center for Interdisciplinary Research, Bielefeld UniversityBielefeld, Germany; ^4^The University of QueenslandSt. Lucia, QLD, Australia

**Keywords:** eye movements, search efficiency, facial expression, attention, visual search

## Abstract

Some targets in visual search are more difficult to find than others. In particular, a target that is similar to the distractors is more difficult to find than a target that is dissimilar to the distractors. Efficiency differences between easy and difficult searches are manifest not only in target-present trials but also in target-absent trials. In fact, even physically identical displays are searched through with different efficiency depending on the searched-for target. Here, we monitored eye movements in search for a target similar to the distractors (difficult search) versus a target dissimilar to the distractors (easy search). We aimed to examine three hypotheses concerning the causes of differential search efficiencies in target-absent trials: (a) distractor dwelling (b) distractor skipping, and (c) distractor revisiting. Reaction times increased with target similarity which is consistent with existing theories and replicates earlier results. Eye movement data indicated guidance in target trials, even though search was very slow. Dwelling, skipping, and revisiting contributed to low search efficiency in difficult search, with dwelling being the strongest factor. It is argued that differences in dwell time account for a large amount of total search time differences.

## Introduction

Visual search is the task of finding a target among non-target distractors. Visual searches vary in difficulty. Some searches are easy and the target is detected at first glance. In other cases, search is difficult and the target is found only after inspecting a number of distractors. Search difficulty is often measured as search efficiency. Search efficiency is a formal characterization of poor and good performance in visual search experiments where the number of non-target stimuli in the display is varied (variation of set size) and response time (RT) for correct decisions on target-absent or target-present is measured. Search efficiency is measured in ms/item, which is given by the slope of the RT/set-size function. Search may be efficient yielding a slope near zero, which means that search time is largely independent of the number of distractors. In other cases, search may be inefficient with a positive slope of the RT/set-size function, indicating that adding more items increases RT. Search efficiency is a continuum, and may assume any number between slightly negative slopes up to more than 100 ms/item per item in target trials (Wolfe, 1998).

It is well-known that target-distractor similarity (Neisser, 1964; Duncan and Humphreys, 1989) impacts strongly on the time needed to find a target among non-target distractors in visual search. When the distractors are dissimilar from the target, search is easy, and when the distractors are similar to the target, search is difficult. This fact is consistent with prominent theories of visual search that hold that target acquisition is governed by the similarity between the target and the search template – the representation of the target – in a process of *guiding attention* (e.g., Duncan, 1981; Wolfe, 1994; Zelinsky, 2008; Olivers et al., 2011).

The guidance of attention by the target is an intuitively appealing explanation of differences in search efficiency. With better guidance, the target is selected earlier, with fewer non-target items being checked, resulting in overall shorter search times. Extreme conditions of easy search, when the target is very dissimilar from the distractors (dissimilar target) and the target matches the target template exclusively, result in a clear activity peak in an attention guiding priority map, and a high signal-to-noise ratio of the guidance signal. A target similar to the distractors (similar target), in contrast, is difficult to find, because many distractors also match the target template, resulting in high activity peaks also for distractors, and a low signal-to-noise ratio of the guidance signal. These targets are difficult to find, and target candidates have to be checked individually. This explanation seems to be the dominant explanation of differences in search efficiency in prominent models of covert attention shifts (e.g., Wolfe, 1994, 2007) and eye movements (Zelinsky, 2008) during visual search.

Guidance is an elegant and computationally feasible concept for explaining differences in search efficiency. However, a target guidance account cannot be directly applied to performance differences in target absent trials. This is because this explanation focusses exclusively on the ability of the target to guide attention (covert or overt) to its position. One might think that performance in target-absent trials should be relatively constant. For example, in a self-terminating serial search, target candidates would be examined until the target is found in target trials, or until no target candidate is left in target absent trials. In reality, however, there are marked differences in search efficiency in target-absent trials. In particular, target-distractor similarity has a strong effect on the speed of correct target-absent judgments.

For example, in a study by Pashler (1987), participants had to search for a target letter C among 0, 1, 3, or 5 similar distractors (letter G) whereas other distractors were dissimilar (letters X and L). The results showed that the increase in RT with the increase of the number of similar distractors was more pronounced on target-absent trials than on target-present trials (denoted in the following as absent and present trials, respectively). This pattern is not restricted to simple geometric stimuli. Differences in search efficiency in absent trials are also obtained with complex and naturalistic stimuli. Horstmann et al. (2012) found that target-distractor similarity also affects target-absent judgments in a search among faces. In their study, participants had to search through photographic face distractors with neutral expression to detect a target that either differed in both emotion and mouth-opening from the distractors (toothy happy or angry targets), or only in emotion (closed-lipped happy or angry targets). In all trials, the same distractors were shown; yet, in those blocks in which the target was more similar to the distractors (closed-lipped targets), participants took much longer to search through the neutral crowds than in those blocks in which the target was dissimilar (toothy targets). Thus, although participants saw the very same displays, performance was different depending on block-wise target-distractor similarity.

From the perspective of target guidance, it is not immediately obvious why performance in absent trials also depends on target-distractor similarity. As there is no target, differences in guidance by that target should not play a role. Therefore, additional assumptions have to be made to account for target-absent performance. In an extension of the well-known Guided Search 2.0 (Wolfe, 1994) model, Chun and Wolfe (1996) suggested that the threshold for selecting a candidate stimulus can be set differently depending on whether the target produces a clear signal on the activation map. When target-distractor similarity is low, there are only few peaks in the activation map that signal the location of possible target(s) across the visual field, and a relatively high threshold can be applied to separate the (high) activation peak of the target from the (lower) activation peaks of the distractors. In contrast, when target-distractor similarity is high, most stimulus positions will show some activation in the activation map, and the activation values are also similar to each other. In this situation, the threshold for the processing of candidate targets must be lowered to avoid missing the target, which leads to examination of all reasonable candidate stimuli. According to Guided Search 2.0, differences in search efficiency in target-absent trials are thus mostly due to distractor skipping.

There is, however, another possibility. Obviously, search efficiency is essentially the product of the number of tested stimuli and the duration of a test. The threshold account by Chun and Wolfe (1996) focuses on the number of tested stimuli and assumes the duration of a test to be constant. This is a widely held assumption in prominent computational models of visual search, which do not explicitly adjust testing times to explain differences in search efficiency (e.g., Wolfe, 1998; Navalpakkam and Itti, 2006; Zelinsky, 2008). In fact, models of visual attention and eye movements – implicitly or explicitly – often assume a fixed processing time per item, and focus rather on the number of (distractor) stimuli attended or fixated as the determinant of the duration of visual search. For example, in simulations of Guided Search 2.0 models it is assumed that it takes 50 ms to attend and examine a stimulus (cf. Chun and Wolfe, 1996; Wolfe, 1998). Corresponding assumptions are made in other models as well, such as Hulleman and Olivers’ (2016) fixation-based account of visual search, which assumes constant fixation duration of 250 ms.

The alternative that search efficiency may be influenced by the duration of testing items is not part of these models and often noted only as a footnote. This may seem surprising, as this *distractor dwelling* account does not appear to be particularly farfetched or controversial. One reason for the reluctance of models of visual search to incorporate distractor dwelling as a variable might be that if distractor dwelling is an important determinant of performance in absent trials, it must also be a determinant of performance in present trials, which seems to be fully accounted for by differences in guidance provided by the target. Thus, if it is acknowledged that distractor dwelling is a determinant of search efficiency, differences in efficiency often cannot be straightforwardly interpreted in terms of differential guidance by the target.

Is there any evidence that distractor dwelling impacts on search efficiency in both present and absent trials? If distractor dwelling is an important factor, then search slopes in present and absent trials should be highly correlated over variations of search difficulty. This was found by Horstmann et al. (2012) in a visual search study where participants searched for emotional target faces that were either similar or dissimilar to the neutral non-target faces. Set size was varied to probe search efficiency, and search was found to be highly inefficient with slopes of the RT/set-size function in the range of 40–110 ms/item in target-present trials and 110–210 ms/item in target-absent trials. Each participant performed four searches, two relatively easy searches with targets more dissimilar from the neutral distractors, and two relatively difficult searches with targets more similar to the neutral distractors. The correlations between target-present and target-absent slopes were very high. Horstmann et al. (2012) concluded from this result that the duration of distractor rejection processes was the most important determinant of search performance in this task. The easy target was found faster not because it better guided attention to its position, but rather because the distractors were more easily rejected (i.e., categorized as distractors). Given that the distractor faces were identical across all conditions, it follows that distractor rejection critically depended on the similarity of the distractors to the target template. The hypothesis is then that the neutral-face distractors are rejected relatively quickly when compared to a dissimilar target template, on the basis of only superficial comparisons with the target template; in contrast, in difficult search, when target template and distractors are dissimilar, distractors have to be examined with more scrutiny (cf. also, Gould, 1967; Becker, 2011). On this account, differences in search efficiency may even be completely due to differences in the time needed to process and test target candidate stimuli. According to a corresponding *distractor dwelling hypothesis*, search efficiency differences in highly inefficient search should at least in part be due to higher processing demands of distractors when target-distractor similarity is high, which should be reflected in longer dwell times on the distractors when the target is similar to the distractors than when it is dissimilar.

The distractor dwelling hypothesis is not without competitors, though. The correlation between present and absent trials search slopes can also be explained differently. On Chun and Wolfe’s (1996) account, attentional guidance explains efficiency differences in present trials, and a variable threshold explains differences in absent trials. This is because the variable threshold for absent trials is set depending on the degree of guidance in present trials. It could be this dependence that causes the correlation between the present and the absent trials. The problem is that a standard visual search paradigm that measures variations of RT over different set sizes cannot distinguish between these possibilities, because RT cannot distinguish between the number of items considered during search and the time spent on processing each of these items. The slope of the RT/set-size function reflects basically the product of the number of tested stimuli and the duration of the test. To resolve this impasse is one main aim of the present study.

In addition, a third hypothesis may be considered. According to the *revisiting hypothesis* (e.g., Humphreys and Müller, 1993), less efficient search in target-absent trials is due to repeated selection of distractors (‘revisiting’). According to this hypothesis, the probability of missing the target after scanning most of the display is higher in difficult search than in an easy search. To reduce the uncertainty, observers may rescan parts of the crowd, with the tendency to do so being proportional to the probability of missing the target. Thus, higher target-distractor similarity leads to lower search efficiency because observers scan some distractors repeatedly on target-absent trials, to ensure that they have not missed the target.

### The Present Study

The aim of the present study is to empirically test whether the three hypotheses described above – the distractor dwelling hypothesis, distractor skipping hypothesis, and distractor revisiting hypothesis, account for search efficiency differences in target-absent trials when the target is more or less similar to the (identical) distractors. We used a simplified version of Horstmann et al.’s (2012) stimuli and task that had shown a strong correlation between search efficiency in target present and absent trials. Specifically, participants in the present study had to search for an emotional target face that was either similar (closed-lipped angry face; ‘distractor-similar target’) or dissimilar (toothy angry face; ‘distractor-dissimilar target’) to the neutral distractor faces. Similar and dissimilar targets were presented in separate blocks such that the target template was constant within a block but varied between blocks. The important independent variables were target presence (whether the target is present or not in a display), similarity (whether the target was similar or dissimilar to the distractors), and stimulus type within the display (whether a stimulus is a distractor or a target in a target trial).

To distinguish between the distractor dwelling, distractor skipping, and distractor revisiting hypotheses, we monitored the participants’ eye movements during visual search. In advance to RT measurements, eye tracking allows not only to observe the net duration of the processes that finally lead to the decision that there is, or is not, a target in the display (as in RT studies), but also which stimuli are gazed at during search, how long, and when. For the purpose of the present study we assume that the fixation positions can be used as a proxy for what is selected for further processing, and that the duration of gaze during fixations (“dwell time”) can be used as a proxy for the time needed to process the selected item(s). Fixations can also be regarded as indicators of attention, as covert attention shifts precede eye movements (e.g., Schneider, 1995; Deubel and Schneider, 1996).

With these assumptions, we can distinguish between the time spent on processing distractors, and other variables that would influence search duration such as the number of visited versus skipped stimuli, the number of revisited stimuli, and the time spent on processing the target (if present).

Specifically, to examine the distractor dwelling hypothesis that distractors are processed with different levels of scrutiny depending on target-distractor similarity, we measured the distractor dwell times, with a particular focus on target-absent trials. Note that a distinctive prediction of this hypothesis is that fixation time differences arise within continuous inspections of the distractor stimulus, not as a summation effect across repeated visits of the distractor stimulus (which would conflate this hypothesis with the revisiting hypothesis; see below). Thus, in the present study, we used first run gaze dwell time as the dependent variable, which is the sum of durations of fixations during the first continuous visit of the gaze on a stimulus.

Second, to examine the distractor skipping hypothesis that search efficiency varies as a function of the number of distractors that are not inspected during search, we measured whether a distractor was gazed at or not, and compared the proportions between the similar and dissimilar target conditions.

Finally, to assess the revisiting hypothesis that lower search efficiency is primarily due to higher rates of repeatedly selecting distractors, we examined the number of discontinuous re-fixations on distractors.

## Materials and Methods

### Participants

Eight men and four women (age between 19 and 34 years, mean = 23 years) participated for a small monetary compensation (€3).

### Stimuli

**FIGURE 1:**
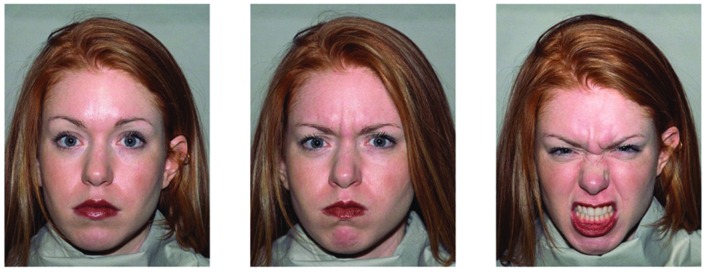
**The same displayer as distractor (Left), similar target (Middle), and dissimilar target (Right)**.

Stimuli were drawn from the NimStim stimulus set (Tottenham et al., 2009). All female models (Displayers 1, 2, 3, 7, 8, cf. Tottenham et al., 2009) provided a neutral face and two variants of angry faces, one with an open mouth and visible teeth (dissimilar target), and one with a closed mouth (similar target, see **Figure 1**). Neutral faces all had a closed mouth. Thus, a total of 15 pictures of faces were used. Each picture subtended 101 × 130 pixels (2.8° × 3.6°), and was coded as a bit map with a color depth of 24 bit (see **Figure 1** for an example of the three expressions that were used from each model).

Search displays consisted of five pictures presented along the diagonals of a 3 × 3 matrix (303 × 390 pixels or 8.4° × 10.8°), that is in the center and in the four corners of the matrix. The target could only ever appear in one of the four peripheral locations (the corners), not in the central location.

### Apparatus

Stimuli were presented on a 19-inch display CRT-monitor (100-Hz refresh rate, resolution 1,024 × 768 pixels) at a distance of 71 cm. A video-based tower-mounted eye tracker (EyeLink 1000, SR Research, Mississauga, Ontario, Canada) with a sampling rate of 1,000 Hz was used for the recording of eye movements. The participants’ head was stabilized by a chin and forehead rest, and in all participants, the right eye was monitored. Before the experiment commenced, the eye tracker was calibrated using a 9-point calibration.

### Design

The experiment comprised two blocks, which differed only in the target category (open versus closed mouth targets, or distractor-dissimilar versus distractor-similar target, respectively). The order of these blocks was counterbalanced across participants. Each block contained 80 trials, 40 of which were target trials, and 40 were absent trials. For each trial, first one of the five models (facial identities) was randomly selected. If the trial was designated as a target trial, this model displayed the target emotion; if the trial was designated as an absent trial, this model showed a neutral expression. Each target face appeared equally often in each of the four possible target positions, which were the outer positions of the 5-stimulus matrix. The remaining four stimulus positions were filled in random order with the remaining four models who all displayed a neutral expression. Thus, on absent trials, search displays consisted of pictures of five different women showing a neutral face. On target trials, one of the outer faces was an angry face (the target). For each target trial, there was a corresponding absent trial where the individual “target” face showed a neutral expression. These “foil targets” corresponded in identity and position to the actual targets. The composition of the displays was computed anew for each trial and participant.

### Procedure

Each trial started with a drift control, where fixation was re-aligned with a central fixation dot once participants pressed a key (with the left hand), which also initiated presentation of the stimulus display. The task was to indicate with a key press (right hand, index or middle finger) whether or not one of the five possible targets was presented in a trial. The search display was presented until the response key press was registered.

Prior to each block (easy vs. hard search), the five possible targets were displayed side by side on the monitor for ad lib inspection, with the aim of providing an overview of their appearance. The experiment started with a 24-trial practice block (not analyzed), presenting 12 target and 12 absent trials with the stimuli from the first task.

## Results

Standard settings of the Eye-Link 1000 were used to parse eye movements into fixations and saccades. Eye movements were parsed as saccades that exceeded an acceleration threshold of 8000 degrees/s^2^, or a velocity threshold of 30 degrees/s. Fixations were inferred from observing that none of these thresholds was exceeded for a period of at least 20 ms.

Interest areas were defined as rectangles of the same sizes as the pictures. For both target and absent trials there were three types of interest areas: (1) the target position that contains either the target in target-present trials or the *foil target* (neutral face of the same person) in target-absent trials; (2) the distractor positions that contained neutral faces drawn from the same pool in both target and absent trials; and (3) the center position that always contained a distractor (and correspondingly, never the target). Analyses were limited to (1) and (2), and excluded (3), because (3) was also the starting point for the search, and for this reason, obligatorily fixated at the beginning of each trial.

Only trials with correct manual responses were analyzed. Manual errors occurred in 4.1% of the trials. An ANOVA on the proportion correct with the variables target presence (target vs. absent trials) and similarity (similar vs. dissimilar) revealed only small effects [target presence: *F*(1,11) = 8.21, *p* = 0.015, $\eta_{p}^{2}$ = 0.184; similarity: *F*(1,11) = 3.3, *p* = 0.096, $\eta_{p}^{2}$ = 0.018; Target presence × Similarity: *F*(1,11) = 4.87, *p* = 0.049, $\eta_{p}^{2}$ = 0.040], reflecting that most errors were misses that occurred more frequently in the difficult search task (see also **Table 1**; Note: Statistical tests are two-sided unless stated otherwise). This pattern of results (more misses than false alarms) is almost universally observed in visual search.

**Table 1:** Mean reaction time (RT), in milliseconds (standard deviations in brackets), and proportion correct (PC) for target present and absent searches for distractor-similar and distractor-dissimilar targets.

		Target absent	Target present
Similar	RT	2265 (546)	1619 (319)
	PC	0.99	0.93
Dissimilar	RT	1673 (441)	1201 (275)
	PC	0.98	0.96

### Manual Reaction Time

An ANOVA of the manual reaction time (RT; 300 < RT < 4,000 ms; 94.2% of the trials) revealed main effects of target presence, *F*(1,11) = 68.17, *p* < 0.001, $\eta_{p}^{2}$ = 0.337, and similarity, *F*(1,11) = 28.76, *p* < 0.001, $\eta_{p}^{2}$ = 0.293. The two-way interaction approached significance, *F*(1,11) = 4.25, *p* = 0.06, $\eta_{p}^{2}$ = 0.012. **Table 1** displays the mean RTs for the four conditions. Mean RTs were longer in absent than in target trials, and longer in the similar than in the dissimilar condition.

### Gaze Dwell Time

**FIGURE 2:**
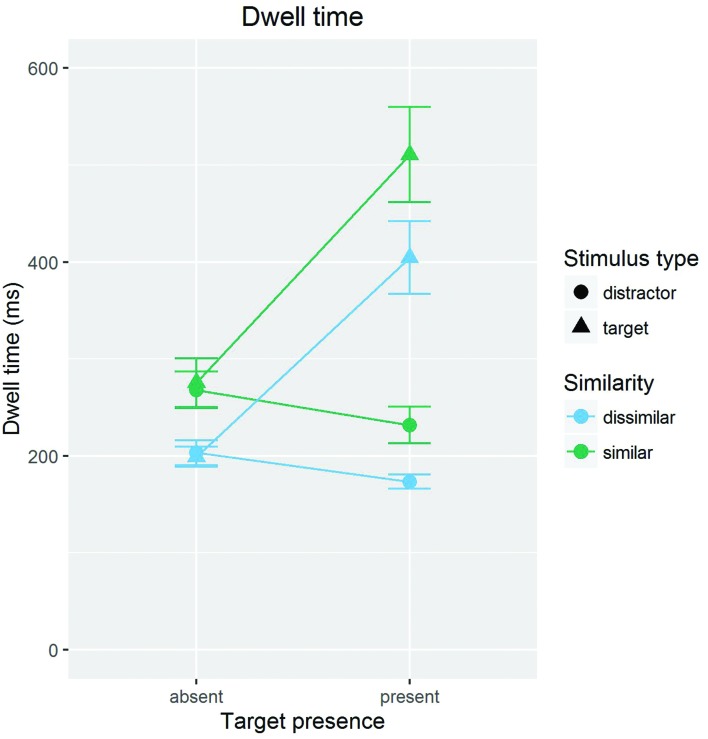
**Mean gaze dwell times, separately shown for targets and distractors of high (similar) and low (dissimilar) target-distractor similarity, in target present and absent trials**. Error bars are standard errors (i.e., *SD*/√*N*) of the means.

To examine the distractor dwelling hypothesis, we analyzed the mean gaze dwell time on the first visit of a stimulus. The same trials were used as for the analysis of RTs. Gaze dwell time is a reasonable summative indicator of the time spent on processing a stimulus, in that it collapses single long fixations and multiple short fixations, as long as these fixations occur within a continuous, uninterrupted inspection of a stimulus. In restricting analyses to the first visit on a stimulus, we can ensure that gaze dwell time is not contaminated by revisiting. Only those data points were included in the analysis where a stimulus was fixated at least once (*N* = 5365); excluding zero duration dwell times ensured that dwell time is not contaminated by skipping. Thirty-three instances of unreasonably short (<40 ms) or long (>2,000 ms) gaze dwell times were excluded from the computation of the means. **Figure 2** gives an overview of the results.

An ANOVA of gaze dwell times with the variables stimulus type (target/foil target vs. distractor), presence (present vs. absent), and similarity (similar vs. dissimilar) revealed main effects for stimulus type, *F*(1,11) = 57.09, *p* < 0.001, $\eta_{p}^{2}$ = 0.353, presence, *F*(1,11) = 50.95, *p* < 0.001, $\eta_{p}^{2}$ = 0.225, and similarity, *F*(1,11) = 18.71, *p* = 0.001, $\eta_{p}^{2}$ = 0.163. These main effects were modified by a significant Presence × Stimulus Type interaction, *F*(1,11) = 64.24, *p* < 0.001, $\eta_{p}^{2}$ = 0.347, and a significant Similarity × Stimulus Type interaction, *F*(1,11) = 6.02, *p* < 0.031, $\eta_{p}^{2}$ = 0.003 (other *F*s < 1.43, *p*s > 0.25).

To specifically test our hypothesis and to clarify the interactions, two ANOVAs were conducted, one for distractors and one for target/foil targets. The ANOVA for distractors with the variables presence (present vs. absent) and similarity (similar vs. dissimilar) revealed main effects for presence, *F*(1,11) = 36.71, *p* < 0.001, $\eta_{p}^{2}$ = 0.096, and similarity, *F*(1,11) = 16.744, *p* = 0.002, $\eta_{p}^{2}$ = 0.270. The interaction was not significant, *F*(1,11) < 1. The main effect for presence is due to longer mean dwell times in absent than in present trials (236 vs. 206 ms). The main effect for similarity reflects longer dwell times on similar than on dissimilar distractors (249 vs. 188 ms).

The ANOVA for targets/foil targets with the same independent variables revealed main effects for presence, *F*(1,11) = 60.19, *p* < 0.001, $\eta_{p}^{2}$ = 0.492, and similarity, *F*(1,11) = 17.67, *p* = 0.002, $\eta_{p}^{2}$ = 0.143. The interaction was not significant, *F*(1,11) = 1.66, *p* = 0.22. The main effect for presence is due to longer dwell times in absent than in present trials (237 vs. 458 ms). The main effect of similarity is due to longer dwell times on similar than on dissimilar distractors (393 vs. 301 ms).

### Indications of Guidance: Target Fixation Latency

**FIGURE 3:**
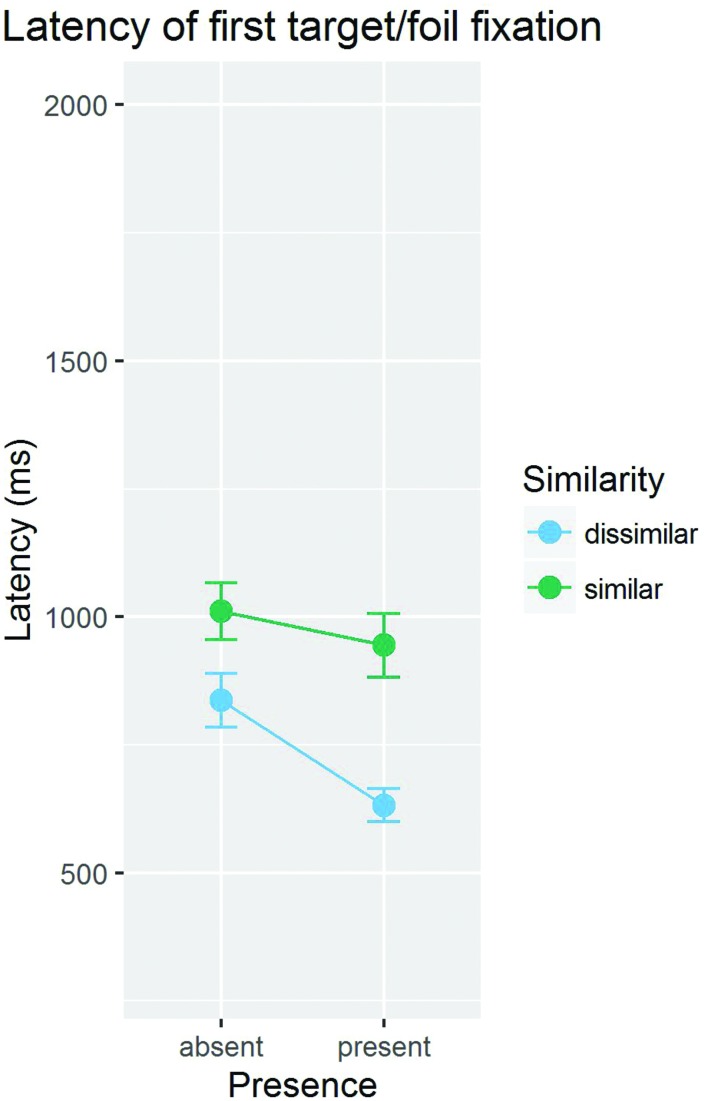
**Mean object selection latencies for the target and the foil target, separately shown for targets similar and dissimilar to the distractors**.

Before we ask how search efficiency in absent trials is determined by distractor skipping, we examine the evidence for attentional guidance by the target in target trials. Conceptually, two questions should be asked with regard to guidance. The first question is whether the target provides any guidance at all, and the second question is whether the easy (distractor-dissimilar) target provides better guidance than the difficult (distractor-similar) target. These questions seem relevant because a previous study with similar stimuli and task had established that searches among photographic faces are highly inefficient (e.g., Horstmann et al., 2012), and because it is not quite clear whether guidance plays a role in highly inefficient search at all (but see Alexander and Zelinsky, 2011). **Figure 3** gives an overview of the mean latencies of stimulus selection for the target and for the foil target.

To address the first question (“Is there any guidance by the target?”), object fixation latencies were compared between target and foil target. Object fixation latencies, defined as the time between the onset of the display and the beginning of the first fixation on a stimulus, were filtered for long (>4,000 ms) and for implausibly short (<100 ms) durations. The results revealed evidence for guidance by the emotional target stimulus: the target was fixated on average 105 ms earlier than the foil target, *t*(11) = 4.60, *p* < 0.001 (pooled over high and low target-similar conditions).

With respect to the second question (“Is guidance stronger for the easy (distractor-dissimilar) than for the difficult (distractor-similar) target?”), we compared the differences in fixation latencies for the target and the foil target across the two conditions. If an easy (distractor-dissimilar) target guides attention better than a difficult (distractor-similar) target, then it should attract fixations earlier than the difficult target. The target was fixated 177 ms earlier than the foil in the distractor-dissimilar target condition, *t*(11) = 4.38, *p* < 0.001, but only 33 ms in the distractor-similar target condition, *t* < 1. The difference in latency advantage was larger in the dissimilar than in the similar condition, *t*(11) = 2.26, *p* = 0.045. In sum, there was guidance by the distractor-dissimilar target, but not by the distractor-similar target.

### Skipping of Distractors in Absent Trials: Is There Evidence for the Adjustable Threshold Hypothesis?

**FIGURE 4:**
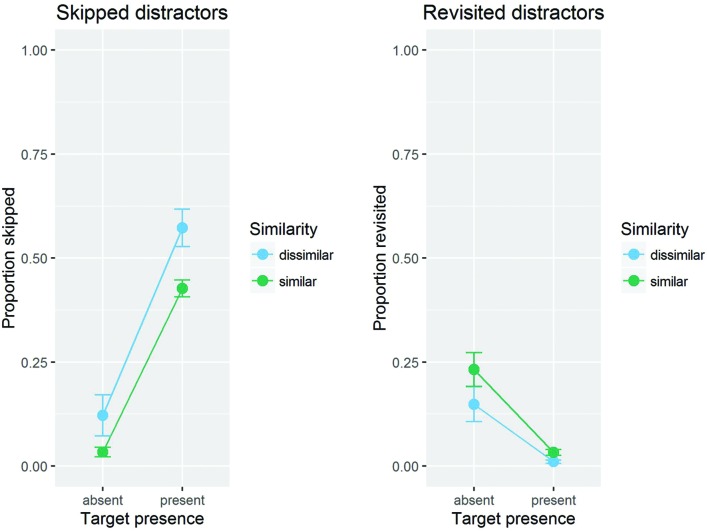
**Mean proportion of skipped distractors (Left), and of revisited distractors (Right), separately shown for present and absent trials, and target-similar and target-dissimilar distractors**. Error bars are standard errors of the means.

To assess whether the threshold for searching through distractors is adapted according to similarity, we computed the probability that distractors were skipped (see **Table 2**, for the results of skipping and revisiting). **Figure 4**, left panel, displays the means for the rates of skipping distractors in overview. On average, skipping rate in absent trials was 0.077 per distractor and trial. To view this figure in context, it translates into 0.318 (0.077 × 4) skipped distractors per trial, because there were always four distractors in an absent trial. Thus, skipping occurred to some degree, indicating that exhaustive search was not implemented perfectly.

**Table 2:** Means (bold), and standard deviations (in brackets) of the proportions of skippings and revisits of a stimulus for distractors in absent and present trials, and for targets in present trials.

		Distractor				Target	
		Absent		Present		Present	
		Dissimilar	Similar	Dissimilar	Similar	Dissimilar	Similar
Skipped							
	*M*	**0.122**	**0.034**	**0.573**	**0.427**	**0.067**	**0.007**
	*SD*	(0.172)	(0.040)	(0.156)	(0.070)	(0.146)	(0.012)
Revisited							
	*M*	**0.148**	**0.232**	**0.010**	**0.033**	**0.157**	**0.269**
	*SD*	(0.145)	(0.142)	(0.013)	(0.025)	(0.134)	(0.199)

Next we tested the effect of similarity. Skipping rate per distractor and trial in absent trials was 0.034 in the similar-target condition and 0.122 in the dissimilar-target condition. Both skipping rates differed significantly from zero, *t*s(11) > 2.46, *p*s < 0.031. The difference between the two difficulty conditions failed to reach the conventional significance level, *t*(11) = 2.1, *p* = 0.055.

### Revisiting: Are There More Runs through the Distractors in Difficult Search?

To assess the hypothesis that increases in search difficulty lead to more frequent revisiting of the distractors, revisiting rate per stimulus was obtained. Revisiting rate was a binary variable that was zero if a stimulus was not visited or only visited once, and one if a stimulus was visited twice or more. **Figure 4**, right panel, displays the revisiting rate for distractors in present and in absent trials.

Our hypothesis concerns revisiting rate on absent trials, which is 0.19 on average per distractor and trial. Revisiting rate was significantly different from zero both in the similar (0.26), and in the dissimilar (0.16) condition, *t*s < 3.54, *p*s < 0.005. Revisiting rate was different for similar and dissimilar targets, *t*(11) = 2.68, *p* = 0.021, with a higher revisiting rate for similar than dissimilar target blocks. Note that a revisiting rate of 0.19 per distractor and trial translates into 0.76 distractors per trial as there were four distractors in a display.

### Correlational Analysis

The data allow for a second variant of analysis. The relative contributions of dwelling, skipping, and revisiting for RT can be examined using a linear regression approach. As before, only the absent trials were of interest. We conducted two analyses. In the first analysis, we analyzed how variance in skipping, dwelling, and revisiting contributed to variance in RTs in general. In a second analysis, we asked how differences in dwelling, skipping, and revisiting account for differences in RT.

**FIGURE 5:**
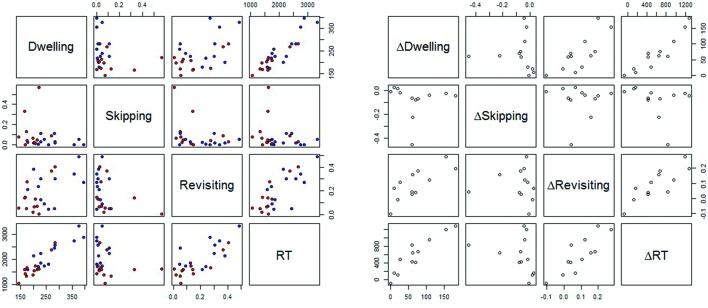
**(Left)** Bivariate relationship between the means of RT, revisiting rate (Revisiting), skipping rate (Skipping), and dwell time (Dwelling), for target absent trials. Each subject contributes two data points, which are the means for dissimilar (red dots) and similar (blue dots) targets. **(Right)** Bivariate relationship between ΔRT, ΔDwelling, ΔSkipping, and ΔRevisiting, respectively, with Δ denoting the difference between the means in the similar and the dissimilar condition.

**Figure 5**, left part, provides an overview of the bivariate relations between the variables RT, revisiting rate, skipping rate and dwell time, for target absent trials (**Table 3**, for the correlation coefficients). In **Figure 5**, each subject contributes two data points, which are their respective means for the dissimilar (red dots) and similar (blue dots) conditions. Two aspects are particularly noteworthy. First, there are clear linear relationships between dwelling and RT, and between revisiting rate and RT. The linear relationship between skipping and RT is somewhat less pronounced. Second, the linear relationship is roughly the same for easy (dissimilar) and for difficult (similar) targets, as red and blue dots align on a single linear function.

**Table 3:** Correlation matrix for the variables similarity, RT, skipping, dwelling, and revisiting.

	Similarity	RT	Skipping	Revisiting
RT	0.53			
Skipping	-0.33	-0.21		
Revisiting	0.33	0.76	-0.33	
Dwelling	0.52	0.91	-0.21	0.53

*Underlined coefficients are statistically significant, p < 0.05.*

We analyzed these data by regressing RT on four variables: dwell time, skipping rate, revisiting rate, and similarity (i.e., coded as “1” for high similarity and “0” for low similarity). The inclusion of similarity in this linear regression equation tests for effects of similarity other than those that similarity has via dwell times, skipping rate, and revisiting rate (see also **Table 4** for the bivariate correlations of similarity with skipping, revisiting, and dwelling).

**Table 4:** Correlation matrix for the differences (similar-dissimilar) in RT, skipping, dwelling, and revisiting.

	RT	Skipping	Revisiting
Skipping	-0.28		
Revisiting	0.84	0.00	
Dwelling	0.94	-0.05	0.78

*Underlined coefficients are statistically significant, p < 0.05.*

There were no indications of substantial collinearity among the predictor variables, with all 1/VIF > 0.56. The regression model rendered a reasonable overall fit with the data, *R*^2^ = 0.94. The standardized beta values for the effects were significant for dwell time, β = 0.69, *p* < 0.001, and for revisiting, β = 0.40, *p* < 0.001. The standardized beta for skipping, β = 0.09, *p* = 0.144, and similarity, β = 0.06, *p* = 0.341, were not significant.

The second analysis targeted the contribution of skipping, revisiting, and dwelling to the similarity effect in our visual search task. More specifically, to account for the similarity effect, the differences in RT (similar-dissimilar) were regressed on the differences (similar-dissimilar) in skipping, revisiting, and dwelling, respectively. **Figure 5**, right part, depicts the main results. **Table 4** presents the correlations. Each point in **Figure 5** corresponds to one participant and represents the difference in the respective variable between the difficult and the easy condition.

There were no indications of substantial collinearity among the predictor variables (all 1/VIF > 0.38). The multiple regression revealed significant effects for all variables and a reasonable fit with the data, *R*^2^ = 0.96. The standardized beta values for the effects were significant for dwell time, β = 0.69, *p* < 0.001, skipping, β = 0.29, *p* = 0.009, and for revisiting, β = -0.24, *p* = 0.030.

## Discussion

The present study examined a number of factors that potentially determine search efficiency differences in absent trials during very inefficient search. Three possible causes for differences in search performance in absent trials were tested: longer dwell times in the difficult task, distractor skipping in the easy task, and revisiting of distractors in the difficult task. Even though all three causes contributed to some degree, the central result is that search difficulty causes sizable differences in dwell times which in turn impact RT, a result that is unexplained by popular models of visual search.

### Dwelling

Our main interest in the present study is the role of dwelling. There were substantial effects of target difficulty on the duration of fixating during the first visit on a stimulus. Gaze dwell times in absent trials were longer in difficult than in easy searches. Moreover, gaze dwell time was strongly correlated with overall search time (RT), and was the strongest predictor of RT in the linear regression. As emphasized at the outset of this paper, this is not particularly surprising from a general analytic point of view. Nonetheless, the adaptation of dwell time does not prominently feature in popular theories of visual search (Wolfe, 1994; Chun and Wolfe, 1996; Navalpakkam and Itti, 2006; Zelinsky, 2008).

It is noteworthy that dwell time differences on distractors were the same for target trials and absent trials. This result directly supports Horstmann et al.’s (2012) suggestion that the efficiency differences in target trials are due to the same processes that are operational in absent trials, and that these are processes of distractor processing (“distractor rejection”). While Horstmann et al. (2012) sought support for that hypothesis in correlations between present and absent search slopes of the RT/set-size function, the present study shows converging evidence by the more direct measurements of gaze data.

Gaze dwell times were much longer on targets than on distractors, but the search difficulty effect was similar. That gaze dwell times were longer for the target may be attributed to the fact that participants remained fixated on the target during response selection, instantiating the general tendency to couple perception and action by attention (e.g., Schneider et al., 2013; Herwig, 2015). That the effect of search difficulty on dwell time was of similar magnitude for target and distractors suggests that the extra time to reject a distractor is roughly the same as the extra time to confirm a target. This has an interesting implication, if one considers dwell time as a covert visual search within a single fixation. To be in agreement with equal effects in present and absent trials, this search has to be exhaustive. This contrasts with self-terminating search which is usually assumed for visual search. It resembles, however, memory search (Sternberg, 1969), where likewise no differences between present and absent trials are found. Therefore, one possible explanation would be that the target representation in working memory was more like a list of features than a holistic template, and that the difficult target representation contained more features than the easy target.

To summarize, there were sizable differences in gaze dwell times between easy and difficult search conditions. These differences were similar for target-absent and target-present trials (i.e., there was no interaction between difficulty and target presence). This result is consistent with the claim that the impact of search difficulty on search efficiency is due to the same process of distractor rejection in target and absent trials.

### Attentional Guidance, Settable Thresholds, and Distractor Skipping

One prominent and principled treatment of search performance in absent trials has been presented by Chun and Wolfe (1996) as an extension to Guided Search 2.0 (Wolfe, 1994). Guided Search 2.0 proposes that a first preattentive stage of processing yields an activation map, which provides the basis for visual selection of objects during the second, attentive stage. In the activation map, bottom-up signals are combined with top-down biases that emerge from expectations about the target and its features (target template). In particular, bottom-up evidence is amplified by top-down information about the expected target features, modulating the peaks in the activation map. The attentional stage then selects objects by following the activation gradient (perseverance is prevented by applying inhibition to previously visited locations). An easy search, for example, resulting from embedding a target among very dissimilar distractors, is characterized by a single distinct activation peak in target trials. In contrast, a difficult search, for example resulting from embedding a target among very similar distractors is characterized by several activation peaks of about equal height, each of which corresponds to one of the objects in the search display. Because there is only one highly probable target candidate in easy search, many distractors are skipped in target trials. Moreover, because of a clear activation peak in target trials, it is possible to set a high activation threshold for the continuation of search in absent trials, such that below-threshold activations (e.g., low activations because of bottom-up saliency signals) can safely be ignored. In contrast, in a difficult search, there is no clear difference between the target and a distractor in their activation values, and the threshold for continuing search must therefore be relatively low. Thus distractor skipping frequency is low.

The present results provide evidence for attentional guidance in target trials with dissimilar targets. Fixation latencies were lower on the target than on the foil target, indicating that the gaze was directed earlier to the target than to the foil target. Moreover, in accordance with Guided Search 2.0, guidance was stronger for dissimilar than for similar targets.

The above mentioned results suggest that the easy (dissimilar) target provided some guidance in the present experiment, in contrast to the difficult (similar) target. This is not a trivial result, given that search for comparable stimuli and tasks was very inefficient in a previous experiment, being around 40 and 80 ms/item for easy and difficult targets, respectively, in target trials (Horstmann et al., 2012). Apparently, very inefficient search and guidance are not mutually exclusive. This is consistent with results from Alexander and Zelinsky (2011), which also showed attentional guidance from complex stimuli in a category search task.

After having established that there was guidance by the target during search in present trials, we tested the more specific differential threshold hypothesis for performance in absent trials. On this account, more distractors are skipped in the easy than in the difficult search condition. This prediction turned out to be true: While skipping in target absent trials was very rare in difficult search, it was more frequent in easy search. That is, parts of the differences in search times induced by search difficulty are apparently due to differences in the frequency of distractor skipping, as predicted on the basis of Chun and Wolfe’s (1996) extension of Guided Search 2.0. This conclusion is also supported by the regression analysis, where the skipping difference between easy and hard search significantly contributed to the RT difference between easy and hard search.

One might register that skipping rate was low in the present experiment, and that the differences in skipping that were induced by search difficulty were likewise low. Thus, in the present search displays, the large difference in search times can hardly be explained in terms of the number of skipped distractors, indicating that a variable threshold does not account for a major part of the differences between easy and difficult search in absent trials.

### Revisiting of Distractors

On average, slightly less than one distractor was revisited every absent trial. More importantly, similarity influenced revisiting. In the difficult task with the distractor-similar target, revisiting rate per trial was higher than in the easy task with the distractor-dissimilar target.

**Figure 4** suggests that revisiting was more frequent in absent than in present trials. This validates the original idea of Humphreys and Müller (1993) that revisiting occurs in particular after an unsuccessful scan, that is, when participants fail to detect the target after a first scan. The regression analysis also revealed substantial correlations between revisiting in search time. Revisiting evidently contributed to the difficulty effect in visual search.

Another reason of why distractors were revisited might be position memory failure – a stimulus is revisited when the information is lost that it had been checked before (e.g., Horowitz and Wolfe, 1998; Peterson et al., 2001). On the one hand, this would not seem to be a very probable explanation in the well-structured display and with the small set size in the present experiment. On the other hand, the extended duration of a single trial might contribute to loss of position memory.

A further plausible reason is that revisiting occurs because the information gathered during the first visit is insufficient for a decision. According to some authors (e.g., Hooge and Erkelens, 1996, 1998), participants plan the time of the next stimulus fixation partly on the basis of the average time needed for a decision in the past. Accordingly, there would be a fraction of trials where the eyes leave the stimulus before a decision has been made, and have to return to the stimulus to resume processing. This depiction somewhat naively assumes that stimuli processing ceases immediately when gaze leaves. More probably is rather that processing continues for some time after the end of the fixation, and that the gaze returns because insufficient sensory information has been accumulated in the first visit. This is in agreement with the observation that revisits often occur after only one or two additional stimuli have been visited (e.g., Gilchrist and Harvey, 2000).

Hulleman and Olivers (2016) recently presented a visual search model in which revisiting is the second important determinant of search efficiency, in addition to the prime determinant, which is the functional field of view (FoV). This model assumes limited avoidance of previously fixated areas due to a limited capacity memory of four items. This model apparently does not explain the differences between easy and hard search in the present study. As the number of items is four in both conditions, the same low number of re-fixations is predicted for both conditions.

### Interaction between the Variables

Assuming for simplicity that saccade duration is negligible, search time is roughly the product of dwell time and the number of visited items plus a constant. This has the consequence that dwell time interacts with skipping and revisiting in their impact on search times. Furthermore, skipping and revisiting are additive, with skipping always having a negative sign and revisiting having a positive sign. For an absent trial, the search time could be formalized as follows:

$$(1)\;search\,\;time=dwell\,\;time\times (number\,\;of\,\;distractors-skipping+revisiting)+{cons\,\tan t}^{1}$$

This leads to an interesting observation. In difficult search, there is little skipping and lots of revisiting, such that the dwell time receives a relatively large weight in determining search time. In contrast, in easy search, there is a lot of skipping and little revisiting, such that dwell times receive a relatively small weight. Dwell times vary in the same direction: These are longer in difficult search and shorter in easy search.

To summarize, search difficulty impacts on two determinants of search time, dwell time and number of items being inspected, that are combined by multiplication. It follows that when search difficulty is varied linearly, then its effect on search time varies more than proportionally.

### Strategic or Situational Variables?

Are the variables discussed here – skipping, revisiting, and dwelling – strategic or situational? Are they set oﬄine, before the trial (cf. Chun and Wolfe, 1996), or online, in response to the very stimuli presented in the display?1With the constant set arbitrarily to zero, this simple model accounts for 87% of the variance in mean absent RTs (*r* = 0.935). Note, however, that the single correlation of dwelling and RT is almost as high (see **Table 3**).

Chun and Wolfe (1996) proposed that skipping rates are determined oﬄine – before the trial – through an adjustable threshold that determines whether an item will be attended or skipped. Dwelling, on the other hand, can be both, situational or strategic. It could be that dwell time is block-wise adapted to search difficulty, anticipating the usual duration of processing necessary to discriminate a target from a similar or dissimilar distractor (e.g., Hooge and Erkelens, 1996, 1998). The other possibility is that dwell time is to a large degree a direct response to the stimulus. When we consider the rejection of a distractor, this seems reasonable: The decision “different” should be easier (and faster) when the search template is clearly dissimilar to the distractor. In line with on-line effects of search difficulty on distractor rejection, Becker (2011) found dwell times on target-similar distractors to be longer than on target-dissimilar distractors when both were present in the same display.

### Search Difficulty, Similarity, and Salience

Is it possible that low-level salience influenced search and may explain the similarity effect? Target-distractor similarity can be regarded as an instance of saliency as the dissimilar target deviates perceptually from the neutral distractors more than the similar target. It is well possible that participants use this characteristic, rather than the target template, for guiding attention to the target. This is known as singleton search mode, where attention is guided by visual saliency during visual search (Bacon and Egeth, 1994). It is also possible that a salient target face would capture attention, independently from the activated target template or singleton search mode, as would be predicted on a saliency capture account (e.g., Itti and Koch, 2000; Theeuwes, 2010). The present data cannot ultimately decide between guidance by salience and guidance by a target template. However, the relatively long reaction times and the inefficiency of the search render a singleton capture account rather improbable. Horstmann et al. (2012), using partly the same stimuli and a similar task, found very inefficient search for all stimuli, even for the targets that were most dissimilar from the distractors. Similarly, in the present experiments, RTs were rather long, as were the target selection latencies. This is opposite of what would be expected if search was based on saliency. In fact, saliency theorists (e.g., Itti and Koch, 2000; Theeuwes, 2010) tend to assume that saliency is relevant for selection very early in time, affecting in particular early attention shifts and first eye movements. That all searches have been very slow would thus suggest that saliency had a weak influence here, if any. It might be noted that a saliency account is also less parsimonious than a target-template account because different representations would be used for guiding attention to a candidate (i.e., saliency), and for testing the candidate against a definition of the target (i.e., the target template).

### Implications for Models of Visual Search

A major motivation for the present research was to test whether the main assumption of popular visual search models is valid: That search efficiency has to be explained by the number of items processed, and not by the time spent on processing. For example, on the model extension of Guided Search 2.0 by Chun and Wolfe (1996), search efficiency in present trials is determined by guidance to the target, and search in absent trials by a variable threshold of search continuation. The present results give reasons to doubt that the variable threshold is the only, or even the most important determiner of visual search performance. Rather, it seems that search performance is determined by both the number of stimuli processed and the duration of stimulus processing. Moreover, this holds true for both target and absent trials. While this is certainly not surprising from an analytic point of view, it changes the interpretation of search efficiency. In Guided Search 2.0, a difference in search slope in a target trial is due to a difference in guidance by the target. If the present results generalize, differences in search slope may be due to any combination of guidance by the target, and dwelling on distractors.

This conclusion cannot be avoided by pointing out that the present stimuli were very complex and search very inefficient. Firstly, it is likely that the kind of search is representative for searches in daily life. In fact, Hulleman and Olivers (2016) recently criticized that visual search studies focused excessively on easy searches, to the neglect of difficult searches. Clearly, a general model of visual search should explain difficult searches as well as easy searches. Secondly, the two search conditions differed in guidance by the target. This result suggests that models that focus on explaining guidance by the target are relevant here.

It might be objected that the present study is about eye movements, while models of visual search such as Guided Search are about covert shifts of attention. This objection, however, is not completely accurate. Models of visual search such as Guided Search are in fact agnostic of whether attention shifts are covert or overt, and they must be: Standard visual search experiments allow both covert and overt shifts of attention, and the measure of RT does not differentiate between these two types.

The present results question interpretations of search slopes in previous research. For example, a large literature on search differences by emotional stimuli assumes that steeper slopes for one category of stimuli (e.g., hostile facial expressions) over another category (e.g., friendly facial expressions) indicate that these faces provide better guidance (e.g., Öhman et al., 2001). In the light of the present results, this implication is no longer credible. Differences in slope may as well be due to longer dwelling on distractors stimuli.

## Conclusion

The study of visual search focuses primarily on the determinants of performance in target trials. While this choice is reasonable as the guidance of attention to the target is a fascinating topic, it has led to a neglect of explaining performance in absent trials. The present research found evidence for three possible causes of differences in search efficiency in absent trials: attentional dwelling, distractor skipping, and distractor revisiting. Not only have these results implications for the interpretation of absent trial performance, but also on target trial performance, because the time spent on the distractors is an important variable in target trials as well. Computational models of attention such as Guided Search 2.0, the Target Acquisition Model, or the Saliency Model, set this time to a constant. The present results imply that distractor processing time is not a constant, but variable and dependent on search difficulty, indicating the need to incorporate this variable in models of visual search.

## Author Contributions

Conceived and designed the experiments: GH. Suggested statistical procedures: GH, AH, and SB. Analyzed the data: GH. Contributed to the interpretation of data for the work: GH, AH, and SB. Drafted the work: GH. Critically revised the work: GH, AH, and SB. Approved the revision to be published: GH, AH, and SB.

## Conflict of Interest Statement

The authors declare that the research was conducted in the absence of any commercial or financial relationships that could be construed as a potential conflict of interest.
